# Gamma Irradiation Resistance of Four Elastomers for Nuclear Sealing Applications

**DOI:** 10.3390/polym18010114

**Published:** 2025-12-30

**Authors:** Xiaohui Du, Caixia Miao, Qi Sun, Haijiang Shi, Hongchen Han, Lili Chu, Guanghui Zhang, Hongchao Pang

**Affiliations:** 1 School of Environmental Science and Engineering Technology, Tianjin University, Tianjin 300354, China; du_ciae@163.com; 2 Department of Nuclear Safety and Environmental Engineering Technology, China Institute of Atomic Energy, Beijing 102413, China; miaocx@ciae.ac.cn (C.M.); sunq@ciae.ac.cn (Q.S.); 18811093853@163.com (H.S.); tjumchan@163.com (H.H.); lij@ciae.ac.cn (L.C.);

**Keywords:** rubber materials, γ-irradiation, radiation resistance, cross-linking, scission

## Abstract

The reliability of rubber materials in nuclear sealing applications depends on their resistance to ionizing radiation. To explicitly reveal the differences in radiation damage mechanisms among rubbers with varying molecular structures, this study investigated four typical elastomers—natural rubber (NR), butyl rubber (IIR), chloroprene rubber (CR), and nitrile rubber (NBR)—under ^60^Co γ-irradiation at cumulative doses of 1, 10, and 100 kGy. By coupling macroscopic physical testing (mechanical, permeability) with microstructural characterization (FT-IR, DSC, crosslink density), a correlation between material structure and irradiation behavior was established. The results indicate that main-chain saturation dictates the dominant degradation mechanism: unsaturated rubbers (NR, CR, NBR) are dominated by cross-linking, macroscopically manifested as increased hardness and reduced ductility; conversely, saturated rubber (IIR) is dominated by main-chain scission, leading to a paste-like transition at 100 kGy and a complete loss of mechanical load-bearing and barrier functions. Comparatively, NR exhibited optimal overall stability due to “clean” cross-linking without significant oxidation. The overall radiation resistance ranking within the 0–100 kGy range is NR > CR > NBR > IIR. This study clarifies the “structure-mechanism-property” evolution law, providing a critical theoretical basis for lifetime prediction and rational material selection of rubber components in nuclear environments.

## 1. Introduction

In fields such as nuclear power generation, radiological medicine, and nuclear technology research, personnel are inevitably exposed to ionizing radiation, posing significant threats to occupational health and safety [1,2,3]. Consequently, the development and application of high-performance radiation protection equipment have become crucial for ensuring the safety of relevant personnel [4,5]. In various protective applications, including protective clothing, gloves, and hoods, materials are required to offer both protective functionality and excellent operational flexibility [6,7,8]. Owing to their irreplaceable flexibility and high resilience, rubber materials are the preferred matrix for manufacturing lightweight, soft, and form-fitting protective systems [9,10]. However, a key challenge that must be addressed is that rubber, as an organic polymer, is generally more sensitive to γ-irradiation and more susceptible to aging compared to inorganic materials like metals or ceramics [11,12]. Given that the rubber matrix commonly functions as the core structural component in protective equipment, often in integration with a functional shielding layer, its stability under radiation conditions plays a decisive role in the overall reliability and service life of the protection system [13,14,15,16].

Cobalt-60 (^60^Co) is a radionuclide with an atomic number of 27 and a mass number of 60. The gamma rays (γ-rays) emitted during its decay possess high penetration capability [17]. As a form of high-energy electromagnetic radiation, γ-rays can cause significant harm to the human body by ionizing or exciting biological macromolecules such as nucleic acids and proteins, thereby altering their structure and function, and disrupting cellular activity and metabolism [18]. ^60^Co γ-irradiation experiments are commonly employed to simulate radiation environments and evaluate the radiation resistance of materials [19]. When γ-rays interact with rubber, processes such as the photoelectric effect, Compton scattering, and electron-positron pair production may occur. Most of the energy is absorbed by the rubber through secondary electrons generated in these interactions [17]. The γ-rays emitted by ^60^Co have an energy of approximately 1.25 MeV, while the rest mass of an electron or positron is about 0.51 MeV [19]. Therefore, the probability of pair production is very low in this context, and energy absorption occurs predominantly through the Compton effect.

When rubber materials are used for extended periods in radiation fields, the absorbed energy can lead to deterioration in their physical and chemical properties [20,21,22]. This degradation manifests as reduced mechanical strength, loss of elasticity, accelerated oxidation, and microstructural damage, ultimately resulting in functional failure. The radiation resistance of rubber materials refers to their ability to retain their inherent physical, chemical, and mechanical properties under specific irradiation conditions. Macroscopic property degradation stems from competing mechanisms of molecular chain scission and cross-linking at the micro-scale induced by irradiation [23,24]. Several studies have explored the irradiation behavior of different elastomers. For example, Luo et al. investigated the properties of nitrile rubber (NBR) under irradiation and observed that increasing the total dose led to greater cross-linking, which in turn reduced mechanical properties, increased hardness, and decreased both tensile strength and elongation at break [25]. Hanada et al. reported that in silicone rubber, irradiation at low temperatures (<100 °C) promoted Si–O–Si cross-linking, enhancing tensile strength [26]. However, under high-temperature (>150 °C) or high-dose (>500 kGy) conditions, chain scission became dominant, resulting in decreased molecular weight, reduced hardness, and increased permanent set. Marinović-Cincović et al. compared ethylene-propylene-diene monomer (EPDM) and chlorosulfonated polyethylene (CSM) elastomers with different curing systems under γ-irradiation [27]. They found that the crosslink density of peroxide-cured EPDM increased nearly threefold after a 200 kGy dose, whereas the sulfur-cured system experienced crosslink network breakdown due to polysulfide bond cleavage. Xiao et al. observed in irradiation tests on fluoroelastomer (FKM) that increasing the dose raised the glass transition temperature (Tg) and compression set, indicating a decline in elastic recovery [28].

Although these studies reveal the performance evolution of specific rubbers under irradiation, the growing complexity of radiation environments and application scenarios highlights the lack of systematic comparative analyses among rubber materials with different structures. This gap makes it difficult to uniformly interpret their irradiation damage mechanisms from a molecular perspective. Therefore, a comprehensive performance evaluation and mechanistic comparison of various rubbers under identical irradiation conditions holds significant theoretical and practical value for engineering applications.

Based on this, this study selected four representative commercial rubber materials—Natural Rubber (NR), Butyl Rubber (IIR), Chloroprene Rubber (CR), and NBR—for a systematic investigation of their performance and structural evolution under uniform ^60^Co γ-ray irradiation conditions. The chemical repeat-unit structures of these four elastomers are as follows:

NR: [-CH_2_-C(CH_3_)=CH-CH_2_-]_n_

IIR: [-CH_2_-C(CH_3_)_2_-]_n_ (primary component) and [-CH_2_-C(CH_3_)=CH-CH_2_-]_n_ (minor component, ≈1–2%)

CR: [-CH_2_-CCl=CH-CH_2_-]_n_

NBR: Copolymer consisting of [-CH_2_-CH=CH-CH_2_-]_n_ (≈70%) and [-CH_2_-CH(CN)-]_n_ (≈30%)

By comprehensively employing macroscopic property testing and microscopic structural characterization, the aims are to: quantitatively compare the differences in radiation resistance among the four rubbers within the 0–100 kGy accumulated dose range; and establish the correlation between their macroscopic property degradation and the microscopic cross-linking/scission mechanisms. This work will provide a crucial theoretical foundation and data support for the rational design, reliability assessment, and safe application of high-performance, long-life rubber protective products in the nuclear industry.

## 2. Experimental

### 2.1. Materials and Instruments

#### 2.1.1. Rubber Materials and Formulations

Source of Vulcanized Compounds: The rubber compounds used in this study were compounded and provided in the uncured state by our collaborator, Beijing Huateng Rubber & Latex Products Co., Ltd., Beijing, China.

Raw Material Specifications and Formulations: NR (Grade 3L, Haining Hongfeng Rubber & Plastic Co., Ltd., Dongying, China), IIR (Grade 1751, Yanshan Petrochemical Company, Beijing, China), CR (Grade CR121, Shanxi Huojia Changhua Synthetic Rubber, Changzhi, China), and NBR (Grade N3305E, Lanzhou Petrochemical Company, Lanzhou, China).

The main compounding ingredients involved in the experiment are as follows. The reinforcing fillers include carbon black N330 (Cabot Corporation, Boston, MA, USA), silica SiO_2_ (Cabot Corporation, Boston, MA, USA), and CaCO_3_ (Jiangxi Bairui Calcium Carbonate Co., Ltd., Gaoan, China); the vulcanization system consists of ZnO (Shandong Xingyuan Zinc Industry Technology Co., Ltd., Zouping, China), stearic acid (Tianjin Kermel Chemical Reagent Co., Ltd., Tianjin, China), sulfur (Weilin New Material Technology Co., Ltd., Puyang, China), accelerator CBS (SI Group, The Woodlands, TX, USA), and resin SP-1045 (SI Group, The Woodlands, TX, USA), MgO (Tianjin Kermel Chemical Reagent Co., Ltd., Tianjin, China); the antiaging system adopts antioxidant 6PPD (Shandong Shangshun Chemical Co., Ltd., Shouguang, China) and antioxidant TMQ (Kemai Chemical Co., Ltd., Tianjin, China); the colorant includes phthalocyanine green (Dongyuan Chemical, Shanghai, China); the processing aids include polyethylene glycol PEG-4000 (Tianjin Kermel Chemical Reagent Co., Ltd., Tianjin, China), paraffin oil (Tianjin Kermel Chemical Reagent Co., Ltd., Tianjin, China), and silane coupling agent Si69 (Zhongyuwohao New Material Co., Ltd., Dongguan, China). All raw materials are of commercially available industrial-grade or analytical-grade specifications. Formulations of rubber compounds are shown in Table 1.

#### 2.1.2. Instruments

The following instruments were employed in this study:

Universal material testing machine, CMT4104, Suzhou Zhuoxu Precision Industry Co., Ltd., Suzhou, China.

Shore A hardness tester, TH200FJ, Beijing Time Ruid Science & Technology Co., Ltd., Beijing, China.

Gas permeability tester, differential pressure method, VAC-V2, Jinan Languang Electromechanical Technology Co., Ltd., Jinan, China.

Water vapor transmission rate tester, WVTR-W6, Jinan Zhongce Electromechanical Equipment Co., Ltd., Jinan, China.

Fourier transform infrared (FT-IR) spectrometer, Model Nicolet 6700, Thermo Fisher Scientific, Waltham, MA, USA.

Differential Scanning Calorimetry (DSC), DSC300 Caliris Classic, NETZSCH Analyzing & Testing, Bayern, Germany.

Electronic balance, Model BSA323S, accuracy of 0.001 g, Sartorius AG, Göttingen, Germany.

### 2.2. Experimental Procedure

#### 2.2.1. Preparation of Rubber Samples

First, the raw rubber is masticated on an open mill to improve its plasticity. Subsequently, compounding agents (activators, fillers, anti-aging agents, vulcanization systems, etc.) are added sequentially for mixing to produce a homogeneous compound rubber. Then, the compound rubber is placed into a mold and vulcanized on a flat vulcanizing machine under specific temperature and pressure conditions. Finally, the vulcanized rubber sheet is removed and left to stand in a standard laboratory environment for more than 24 h to stabilize its cross-linked network, after which it is cut into standard samples for testing.

#### 2.2.2. Sample Irradiation

Utilizing the experimental facilities of the China Institute of Atomic Energy, the rubber samples (0.8 mm thick) were subjected to irradiation treatment using a ^60^Co γ-ray source. The absorbed dose rate was maintained at 1.0 kGy/h, and irradiation was performed at ambient temperature (≈25 °C) in air. Different accumulated doses were set at 1 kGy, 10 kGy, and 100 kGy to simulate the effects of varying radiation intensity environments on the material properties. Consequently, the respective irradiation times were 1, 10, and 100 h.

#### 2.2.3. Density Measurement

The density of the samples was determined using the Archimedes’ water displacement method. First, the mass of the rubber sample in air (m_1_) was weighed using a balance. Then, the sample was fully immersed in distilled water, and its apparent mass in water (m_2_) was measured. The density of rubber was calculated using the formula:

ρ_rubber_ = m_1_ × ρ_water_/(m_1_ − m_2_), (1)

#### 2.2.4. Water Vapor Permeability Test

The water vapor permeability test was conducted according to GB/T 1037-2021 [29], using the gravimetric method. After cutting the samples using a standard die, the specimen was placed in a test dish containing a desiccant. The dish was assembled and sealed tightly. The water vapor transmission rate was then measured using a Water Vapor Transmission Rate Tester.

#### 2.2.5. Gas Permeability Test

The gas permeability test was performed based on GB/T 7755.1-2018 [30], employing the constant volume method. At room temperature, the rubber sample was mounted in the permeability test cell, creating a high-pressure and a low-pressure side. The low-pressure side was evacuated using a vacuum pump to ensure the sample closely adhered to the support. Air was then introduced into the high-pressure side to reach a test pressure of 300 kPa. The pressure change on the low-pressure side was recorded, and the gas permeability coefficient was calculated.

#### 2.2.6. Mechanical Test

**Figure 1:**
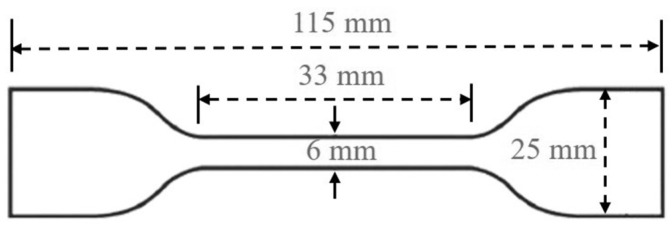
Sample geometry.

Mechanical property testing included tensile tests and hardness measurements. The tensile test was conducted according to GB/T 528-2009 on a universal material testing machine [31]. Dumbbell-shaped standard specimens with a length of 115 mm were used, as shown in Figure 1, with a crosshead speed set at 200 mm/min. Each sample was tested three times, and the average value was taken as the final result. Hardness was measured using the Shore A hardness method, equipped with a spherical indenter. Similarly, each sample was tested three times, and the average value was recorded.

#### 2.2.7. FT-IR Spectroscopy

FT-IR spectroscopy was employed to analyze changes in the surface functional groups of the rubber materials. The test was performed using ATR mode with a scanning range of 4000 to 600 cm^−1^ to detect changes in the chemical structure of the samples before and after irradiation.

#### 2.2.8. Thermal Property Test

Thermal property analysis was carried out by DSC according to GB/T 29611-2013 [32]. A sample weighing approximately 5 mg was placed in a crucible. The test temperature range was from −90 °C to 40 °C, with a heating rate of 10 °C/min. N_2_ was used as the purge gas throughout the test to prevent interference from oxygen.

#### 2.2.9. Equilibrium Swelling Test

Rubber samples with dimensions of 2 cm × 2 cm × 0.8 mm were cut, and their initial mass (W_0_) was accurately weighed. The samples were then completely immersed in a sealed glass container filled with an adequate amount of CH_2_Cl_2_, and stored in the dark at room temperature for the swelling reaction. The mass of the swollen samples (W_t_) was measured every 1 h. For each measurement, the samples were carefully removed, the residual solvent on the surface was quickly blotted dry with filter paper, and the samples were immediately placed into weighing bottles, sealed tightly, and weighed. Subsequently, the samples were put back into the solvent to continue swelling. The swelling process was considered to reach equilibrium when the mass difference between two consecutive measurements was less than 1 mg, and the final equilibrium swelling mass (Weq) was recorded at this stage.

## 3. Results and Discussion

### 3.1. Changes in Macroscopic Physical Properties

#### 3.1.1. Visual Changes

**Figure 2:**
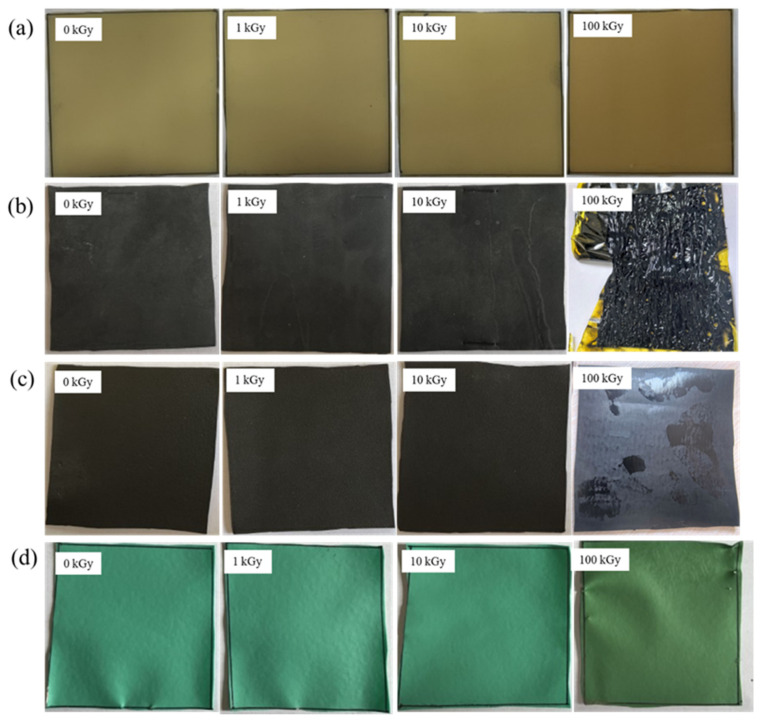
Visual appearance of rubber samples before and after irradiation: (**a**) NR, (**b**) IIR, (**c**) CR, (**d**) NBR.

Images of the four rubber samples before and after irradiation by the ^60^Co γ-ray source are shown in Figure 2 below. Visual observation reveals that irradiation at different accumulated doses affected the appearance of the rubber samples to varying degrees. Under low-dose irradiation conditions (1 kGy and 10 kGy), the appearance of the four rubber samples remained largely unchanged, with no significant color variation or morphological alterations. This indicates that the surface structure of the rubber materials remains relatively stable under lower doses of γ-ray irradiation, allowing them to retain their original characteristics reasonably well. However, when the accumulated dose increased to 100 kGy, significant visual changes occurred in all four rubber samples. Specifically, NR samples exhibited slight darkening in color, potentially due to oxidation caused by molecular chain cross-linking reactions induced by irradiation. IIR samples underwent failure at the high accumulated dose, transitioning to a paste-like state. This suggests severe disruption of its molecular structure under high-dose irradiation, leading to the loss of its original solid form and mechanical properties.CR samples became softer, with an oily substance exuding from the surface. This is likely due to irradiation-induced chemical reactions causing the decomposition and migration of certain components within the rubber to the surface, indicating poor chemical stability of this rubber under high-dose irradiation. The NBR samples exhibited slight yellowing at an irradiation dose of 100 kGy. The color change may result from irradiation-induced oxidation or cross-linking, while the increased hardness is likely due to enhanced crosslink density, reducing the material’s flexibility [33].

#### 3.1.2. Density Changes

**Figure 3:**
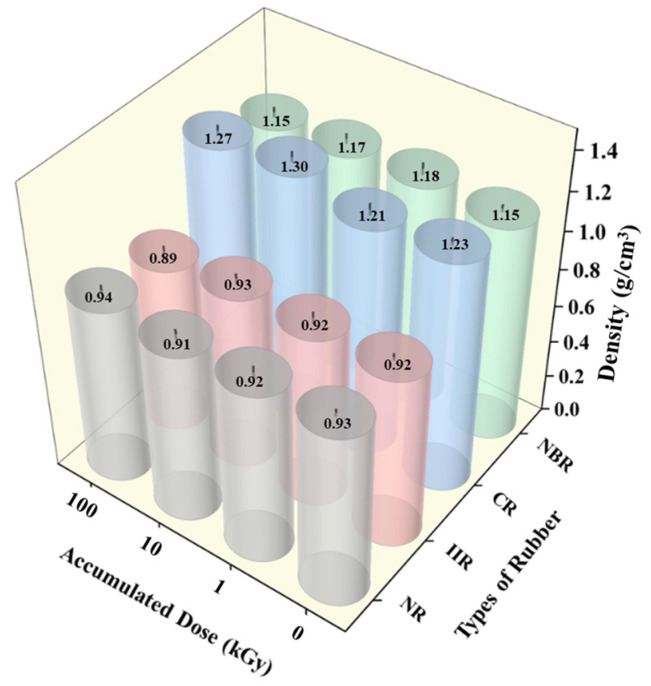
Density changes in the rubber samples before and after irradiation.

The density changes in the rubber samples before and after irradiation are shown in Figure 3. As can be seen, the variation in density for all materials before and after irradiation was relatively limited, not exceeding 8%. With increasing accumulated dose, the density of NR samples remained essentially unchanged. Both CR samples and NBR samples exhibited a slight initial increase in density followed by a more pronounced decrease. The density of IIR gradually decreased.

The macro-density of rubber materials is a comprehensive manifestation of the competition among various microscopic mechanisms. At relatively low irradiation doses where crosslinking dominates, the formation of new bonds between molecular chains results in tighter chain packing and reduced free volume, leading to an increase in density. In contrast, at higher doses or under conditions favoring degradation, main-chain scission occurs, generating low-molecular-weight volatiles and leaving behind micropores. This increase in free volume causes the density to decrease. Some scholars have also reported that the density of semi-crystalline materials depends partly on their crystalline microstructure [34,35]. In the context of this study, the specific factors responsible for the observed density changes need to be identified through subsequent analyses.

#### 3.1.3. Effect of Irradiation on Water Vapor and Gas Permeability

**Figure 4:**
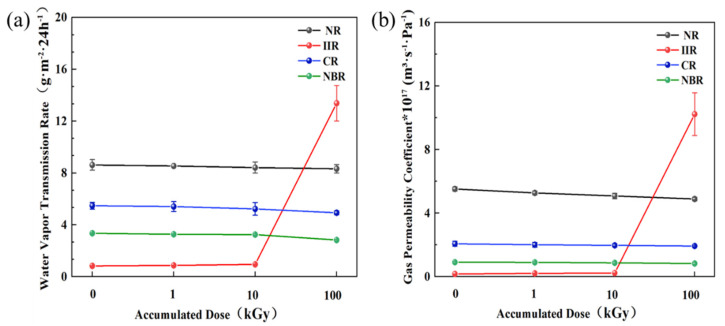
Effect of accumulated dose on the permeability of rubber samples: (**a**) Water vapor transmission rate; (**b**) Gas permeability coefficient.

The permeability test results for the four types of rubber samples under different accumulated doses are shown in Figure 4. The results indicate that irradiation significantly affects the water vapor permeability and gas permeability of the rubber materials. With increasing accumulated dose, the water vapor transmission rate and gas permeability coefficient of NR, CR, and NBR samples decreased. This is a typical consequence of increased crosslink density, which reduces the free volume available for the diffusion of permeants. However, the water vapor transmission rate and gas permeability coefficient of IIR samples increased dramatically after 100 kGy irradiation, by 16 times and 60 times, respectively. This is primarily attributed to irradiation-induced chain scission creating pores, making it easier for water molecules and gases to permeate the material. This phenomenon is highly consistent with the paste-like state observed in IIR samples, where high-dose irradiation caused it to lose its fundamental function as a barrier material. Based on the experimental data, IIR exhibits the best barrier properties against water vapor and gases, followed by CR and NBR, with NR being the least effective. Using this as a reference, IIR can be selected for sealing and protection in products sensitive to water vapor and gas transmission, whereas NR might be suitable for applications where high water/gas barrier performance is not critical.

### 3.2. Evolution of Mechanical Properties

Tensile strength at break, elongation at break, and hardness were used as evaluation indices for the mechanical properties of the four rubber materials before and after irradiation. Tensile strength reflects the maximum stress endured by the rubber material during stretching until fracture, calculated using Equation (2):

Rm = F/S,(2)

where Rm is the tensile strength in MPa, F is the maximum tensile force in N, and S is the original cross-sectional area of the specimen in m^2^.

Elongation at break refers to the percentage increase in length of the rubber material at break relative to its original length, calculated using Equation (3):

ε = (L − L_0_)/L_0_, (3)

where ε is the elongation at break in %, L_0_ is the original gauge length in mm, and L is the length at break in mm. Hardness quantifies the material’s resistance to indentation, which is a macroscopic reflection of its elastic modulus and crosslink density.

**Figure 5:**
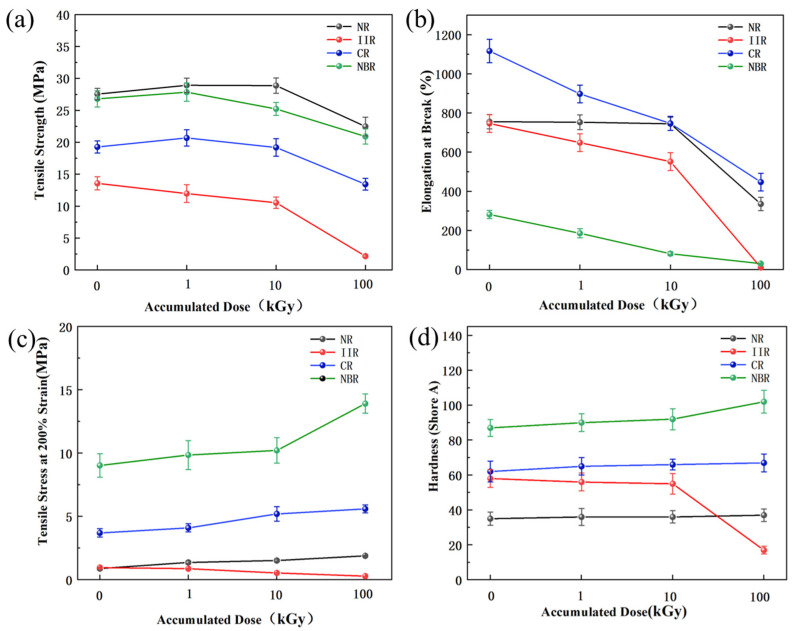
Effect of accumulated dose on the mechanical properties of rubber samples: (**a**) Tensile strength; (**b**) Elongation at break; (**c**) Tensile stress at 200% elongation; (**d**) Hardness.

Figure 5 shows the relationship between accumulated dose and mechanical properties. From Figure 5a, the ranking of tensile strength for the four types of rubber samples both before and after irradiation was NR > NBR > CR > IIR. The tensile strength of NR, CR, and NBR initially increased and then decreased with increasing cumulative accumulated dose. This is because, within a certain dose range, radiation-induced vulcanization promotes increased cross-linking of rubber molecular chains, facilitating stress transfer and distribution; beyond a threshold dose, chain scission and structural damage lead to strength degradation [5]. In contrast, the tensile strength of IIR samples continuously decreased with increasing accumulated dose, indicating direct molecular chain scission under irradiation.

From Figure 5b, before irradiation, the ranking of elongation for the four types of rubber samples at break was CR > NR > IIR > NBR, indicating CR samples had the best longitudinal tolerance and plasticity, with an elongation at break of 1116.54% ± 60.27%. The elongation at break for all four rubbers decreased with increasing accumulated dose.

Figure 5c shows that NBR samples exhibited the highest resistance to deformation, greater rigidity, and a higher elastic modulus at this specific strain level, while IIR samples were the weakest; CR and NR samples were intermediate. The ranking for tensile stress at 200% elongation was NBR > CR > NR > IIR. NR, CR, and NBR samples all showed an increasing trend with dose, with NBR samples having the steepest increase, followed by NR samples, and CR samples showing a relatively gentler but still noticeable increase. IIR samples uniquely exhibited a decreasing trend, contrasting sharply with the others.

From Figure 5d, the initial hardness ranking was NBR > CR > IIR > NR. This hierarchy originates from the intrinsic molecular structures and intermolecular forces of the polymers, which fundamentally govern chain mobility. The exceptionally high initial hardness of NBR is primarily attributed to the strong dipole–dipole interactions induced by the polar cyano groups along its molecular chains. These powerful forces severely restrict chain segment movement. CR ranks second due to the moderate polarity of its chlorine atoms. Although non-polar, IIR exhibits higher hardness than NR because of the significant steric hindrance presented by its densely packed methyl side groups, which impedes chain motion. In contrast, NR, with its non-polar structure and minimal steric hindrance, possesses the most flexible chains, resulting in the lowest initial hardness. High-dose irradiation of 100 kGy caused a sharp decrease in the hardness of IIR samples. The hardness ranking of the other three rubbers after irradiation became NBR > CR > NR. The hardness of NR, CR, and NBR samples increased after irradiation, with NBR samples showing the most significant increase at 100 kGy. IIR samples, however, showed a decreasing trend in hardness.

### 3.3. Analysis of Chemical Structure and Thermal Properties

#### 3.3.1. Effect of Irradiation on Molecular Structure

**Figure 6:**
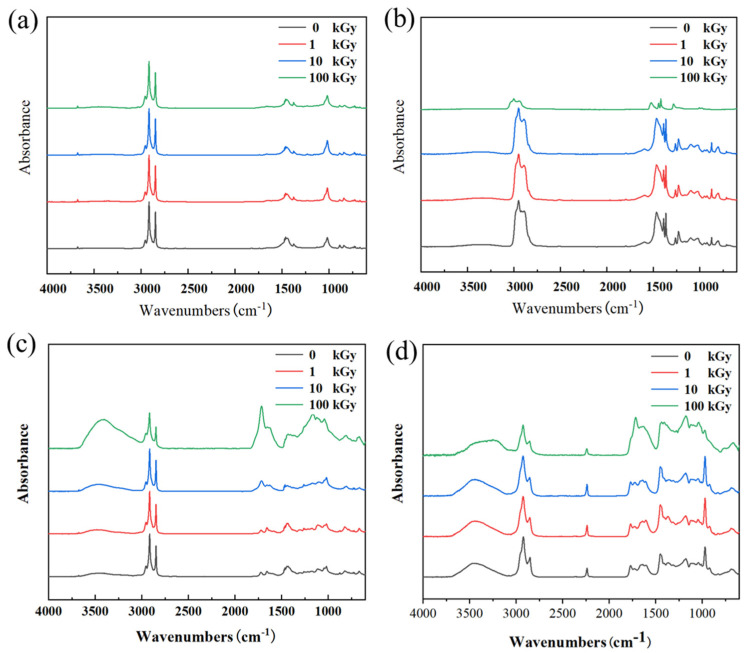
FT-IR spectra of the four rubber samples before and after irradiation: (**a**) NR; (**b**) IIR; (**c**) CR; (**d**) NBR.

Regarding material molecular structure characterization, the FT-IR analysis results are shown in Figure 6. The spectral changes after irradiation revealed significant differences in the degradation mechanisms among the different rubbers. For NR samples, the position and intensity of their characteristic absorption peaks showed no obvious changes before and after irradiation, indicating a relatively stable molecular structure during irradiation. This is primarily because the NR molecular backbone consists of pure carbon-carbon chains without polar or easily oxidizable groups. During irradiation, NR primarily undergoes cross-linking reactions dominated by carbon-carbon bonds, a process that does not introduce new IR-active functional groups; hence no significant changes are observed in the FT-IR spectra. This “clean” cross-linking allows it to harden while maintaining structural integrity. In contrast, after high-dose irradiation of 100 kGy, the intensity of characteristic absorption peaks related to C–H bonds in IIR generally decreased. This indicates that IIR underwent degradation behavior dominated by main chain scission. Chain breakage leads to a decrease in polymer molecular weight and the volatilization of some small molecule products, manifesting as a systematic reduction in C–H vibration signals in FT-IR and corresponding macroscopically to material softening, strength loss, and loss of elasticity. CR exhibited significant structural changes after irradiation: after 100 kGy, the O–H stretching vibration peak at ~3460 cm^−1^, the C=C stretching vibration peak at ~1660 cm^−1^, and the C–O or chain vibration peak at ~1117 cm^−1^ were all significantly enhanced; absorptions near 1400 cm^−1^ and 1000 cm^−1^ also intensified. These changes are attributed to dehydrochlorination reactions in CR under high-dose γ-irradiation, accompanied by the formation of conjugated olefin structures and further oxidation. This series of reactions leads to the accumulation of conjugated double bonds and the generation of numerous oxygen-containing functional groups, indicating that its degradation is a complex process centered on dechlorination, accompanied by oxidation and cross-linking, ultimately causing severe deterioration of physical properties. The aging of NBR under irradiation is primarily dominated by oxidative degradation. With increasing absorbed dose, the absorption peaks observed at ~3450 cm^−1^ and ~1720 cm^−1^ intensified simultaneously, which is a typical characteristic of free radical chain oxidation reactions in polymers. Alkyl radicals generated by irradiation react with environmental oxygen, forming hydroperoxides; these unstable intermediates further decompose, ultimately yielding a range of oxygenated products such as alcohols, ketones, aldehydes, and carboxylic acids. Among these, alcohols and carboxylic acids contribute to the hydroxyl absorption peak, while ketones, aldehydes, and carboxylic acids contribute to the carbonyl absorption peak. The generation and accumulation of these oxygen-containing groups disrupt the original molecular chain structure of the polymer, leading to increased crosslink density and reduced chain mobility, manifesting macroscopically as material hardening, embrittlement, and mechanical property degradation.

#### 3.3.2. Thermal Characterization

**Figure 7:**
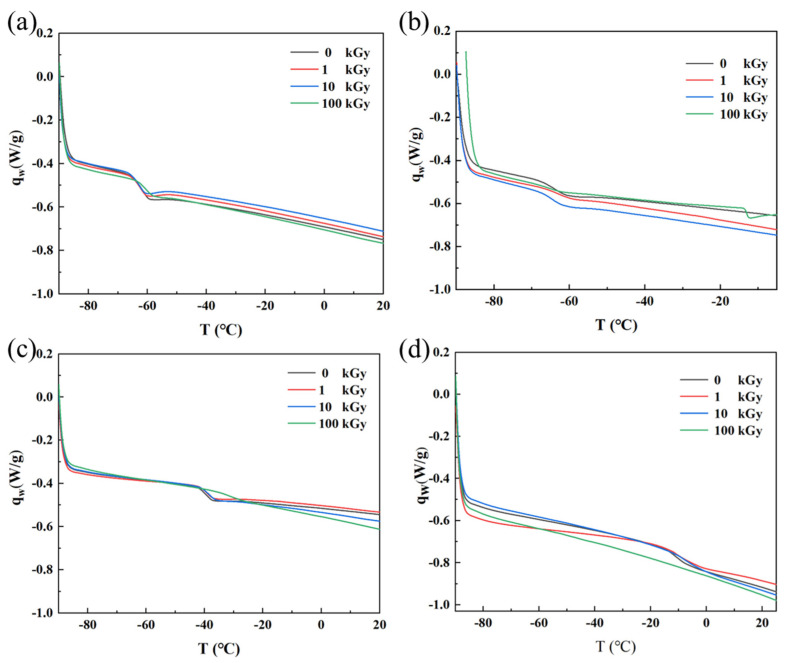
DSC curves of the four rubber samples before and after irradiation: (**a**) NR; (**b**) IIR; (**c**) CR; (**d**) NBR.

The DSC curves of rubber materials reflect changes in the molecular chain motion state across different temperature ranges. Tg marks the transition of the material from a glassy state to a rubbery state, appearing as a distinct step-like change in the curve. A higher Tg indicates better resistance to thermal deformation. Figure 7 shows the DSC curves of the four rubbers before and after irradiation; their Tg values, determined from the inflection points, are listed in Table 2. From Figure 7a and Table 2, the Tg of NR remained essentially unchanged after irradiation, staying around −61 °C. This is because NR undergoes ‘clean’ cross-linking without the generation of strong polar groups, keeping Tg stable. From Figure 7b and Table 2, the Tg of IIR after irradiation at doses up to 10 kGy remained concentrated around −63 °C; after 100 kGy irradiation, no distinct Tg could be detected, proving the loss of typical polymer viscoelasticity under high dose, indicating a significant alteration in the material’s thermodynamic properties. From Figure 7c and Table 2, the Tg of CR after irradiation at doses up to 10 kGy remained near −39 °C, but increased to −31.48 °C after 100 kGy irradiation. This is because irradiation induces cross-linking reactions between CR molecular chains; the increased number of cross-linking points restricts molecular chain motion, making chain segment movement more difficult at lower temperatures and requiring a higher temperature for initiation, manifesting macroscopically as an increased Tg. The experimental results show that the Tg of NBR in its initial state and after accumulated doses of 1 kGy, 10 kGy, and 100 kGy exhibited a trend of increasing with accumulated dose (Figure 7d and Table 2). This is because after irradiation at certain doses, the crystallinity of NBR may change. Research indicates that with increasing accumulated dose, the crystallinity of NBR increases, possibly due to irradiation-induced rearrangement of molecular chains, allowing more chains to arrange in an ordered manner, forming crystalline regions. An increase in crystallinity leads to a rise in Tg [6].

### 3.4. Crosslink Density Analysis by Equilibrium Swelling

To further quantitatively elucidate the degradation mechanisms induced by γ-irradiation, the changes in crosslink density were determined using the equilibrium swelling method. The crosslink densities of the samples before and after irradiation were calculated via the Flory-Rehner equation. The calculation formula is as follows:

(4) v=ln1−V2+V2+xV22V1(−V213+2fV2)

where *v* is the crosslink density, *V*_1_ is the molar volume of CH_2_Cl_2_ (64.0 cm^3^/mol), χ is the polymer-solvent interaction parameter, *V*_2_ is the volume fraction of rubber in the swollen gel, and f = 4 is the functionality of the crosslinks. The interaction parameters χ for the four rubbers in CH_2_Cl_2_ are: NR 0.437, IIR 0.579, CR 0.533, and NBR 0.314 [36].

**Figure 8:**
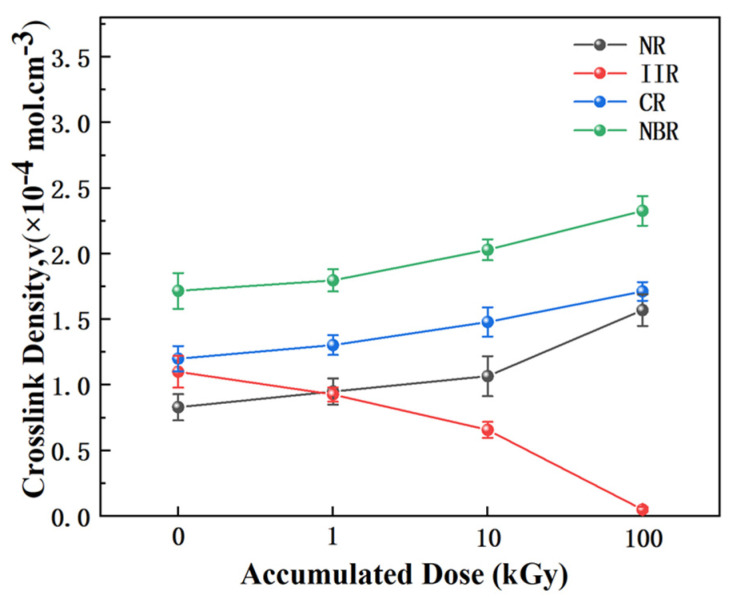
Crosslink density changes in the rubber samples before and after irradiation.

The measured changes in crosslink density provide the most direct quantitative evidence for understanding the radiation degradation mechanisms of the different rubbers. As shown in Figure 8, the crosslink density of NR increased significantly from 0.83 × 10^−5^ mol/cm^3^ (0 kGy) to 1.37 × 10^−5^ mol/cm^3^ (100 kGy) with increasing irradiation dose, representing an increase of approximately 65%. This quantitatively confirms the efficient, “crosslinking-dominated” reinforcement mechanism guided by its unsaturated structure. In stark contrast, the crosslink density of IIR decreased drastically by about 95% to 0.05 × 10^−5^ mol/cm^3^ at 100 kGy. This clearly reveals the network collapse process in its saturated backbone under irradiation, which is “chain scission-dominated”. The crosslink densities of CR and NBR also show a clear increasing trend, indicating that crosslinking remains the dominant reaction for them. However, their increases are smaller than that of NR, which is consistent with the competitive side reactions present in their molecular structures. This further confirms their intermediate radiation stability, positioned between that of NR and IIR. In summary, these quantitative data clearly reveal the distinct evolutionary pathways of the different rubber molecular structures under γ-irradiation, strongly supporting the core thesis of this paper regarding the “structure-mechanism-property” relationship.

### 3.5. Comprehensive Discussion

The experimental results clearly reveal the dominant roles of the two competing mechanisms, cross-linking and chain scission, during irradiation. Due to its unsaturated backbone structure, NR primarily underwent cross-linking, resulting in the best performance retention. IIR, as a saturated rubber, experienced predominantly main chain scission, leading to a comprehensive loss of properties. Although both CR and NBR were dominated by cross-linking, they were accompanied by dehydrochlorination/oxidation and strong free radical oxidation, respectively, resulting in intermediate degrees of performance degradation. Overall, within the 0–100 kGy dose range, the ranking of radiation resistance for the four rubbers is: NR > CR > NBR > IIR.

## 4. Conclusions

This study systematically evaluated the performance evolution and structural change mechanisms of four rubber materials under γ-irradiation, leading to the following conclusions:

1. The radiation damage mechanism of rubber materials is intrinsically linked to their main-chain molecular structure. This study demonstrates that unsaturated rubbers (NR, CR, NBR) are dominated by cross-linking reactions under gamma irradiation, whereas the saturated rubber (IIR) primarily undergoes main-chain scission as its degradation pathway. This fundamental difference dictates the macroscopic performance evolution in a radiation environment: cross-linking leads to material hardening and reduced elongation, while chain scission results in softening or even fluidization.
2. Within the 0–100 kGy cumulative dose range, the four elastomers exhibit significant divergence in their overall radiation resistance, ranked as follows: NR > CR > NBR > IIR. NR displays the optimal stability; its irradiation primarily induces “clean” cross-linking without introducing significant oxidative or polar groups, thereby maintaining high stability in both mechanical properties and Tg. Conversely, IIR is highly sensitive to radiation, suffering severe main-chain scission that leads to a loss of solid form and a complete failure of mechanical and barrier properties at the 100 kGy dose.
3. CR and NBR possess intermediate radiation resistance. While both are dominated by cross-linking, their performance degradation is accompanied by significant secondary chemical reactions. The degradation of CR involves dehydrochlorination and oxidation, whereas NBR exhibits severe free-radical oxidative degradation, leading to the formation of oxygen-containing functional groups that cause material hardening and embrittlement. Consequently, for material selection in nuclear applications, NR is the preferred choice for maintaining flexibility and durability in medium-to-high dose environments, while the suitability of CR and NBR must be strictly evaluated based on their specific oxidative degradation thresholds.

## Figures and Tables

**Table 1:** Formulations of rubber compounds.

Rubber Type	Function	NR	IIR	CR	NBR
Polymer	Matrix	100	100	100	100
Carbon Black N330	Reinforcement	-	50	40	-
Silica SiO_2_	Reinforcement	40	-	-	40
CaCO_3_	Filling	20	-	-	20
ZnO	Activator	5	5	5	5
Stearic Acid	Activator	2	2	0.5	1
Sulfur	Curing Agent	2.5	-	-	1.5
Accelerator CBS	Accelerator	1.2	-	-	-
Resin SP-1045	Curing Agent	-	7	-	-
MgO	Curing Stabilizer	-	-	4	
Antioxidant TMQ	Antioxidation	1.5	1.5	1.5	1.5
Antioxidant 6PPD	Antiozonant	1	1	1	1
Phthalocyanine Green	Colorant	-	-	-	0.25
Polyethylene Glycol PEG-4000	Silica Dispersant	3	-	-	3
Paraffin Oil	Plasticizer	5	5	5	5
Silane Coupling Agent Si69	Tackifier	1	-	-	-

**Table 2:** Tg of the rubbers under different accumulated doses.

Rubber Type	0 kGy	1 kGy	10 kGy	100 kGy
NR	−61.54 °C	−62.18 °C	−61.92 °C	−60.60 °C
IIR	−63.93 °C	−63.19 °C	−64.75 °C	−65.92 °C
CR	−39.24 °C	−39.14 °C	−38.53 °C	−31.48 °C
NBR	−10.78 °C	−11.19 °C	−8.15 °C	−7.47 °C

## Data Availability

The original contributions presented in this study are included in the article. Further inquiries can be directed to the corresponding author.

## References

[1] Lee Y., Choi Y.Y., Yang M., Jin Y.W., Seong K.M. (2021). Risk perception of radiation emergency medical staff on low-dose radiation exposure: Knowledge is a critical factor. J. Environ. Radioact..

[2] Kim S., Lee H.Y., Song J.S. (2018). A study on characteristics and internal exposure evaluation of radioactive aerosols during pipe cutting in decommissioning of nuclear power plant. Nucl. Eng. Technol..

[3] Ohira T., Takahashi H., Yasumura S., Ohtsuru A., Midorikawa S., Suzuki S., Matsuzuka T., Shimura H., Ishikawa T., Sakai A. (2018). Associations between childhood thyroid cancer and external radiation dose after the fukushima daiichi nuclear power plant accident. Epidemiology.

[4] Wu S., Zhang W., Yang Y. (2024). Progress in flexible and wearable lead-free polymer composites for Radiation Protection. Polymers.

[5] Chang Q., Guo S., Zhang X. (2023). Radiation shielding polymer composites: Ray-interaction mechanism, structural design, manufacture and biomedical applications. Mater. Des..

[6] Hao F., Zhou W., Gao Y. (2021). Recent advances in nuclear radiation protective clothing materials. Mater. Express.

[7] Yuan S., Li S., Zhu J., Tang Y. (2021). Additive manufacturing of polymeric composites from material processing to structural design. Compos. Part B Eng..

[8] Elsafi M., El-Nahal M.A., Mohamed A., Ahmed F., Sayyed M.I., Saleh I. (2023). Development of gloves and protective jackets from ionizing radiation made of silicone rubber using nano-bismuth and tin oxides. Silicon.

[9] Li H., Yan L., Zhou J., Wang Y., Liao X., Shi B. (2024). Flexible and wearable functional materials for ionizing radiation protection: A perspective review. Chem. Eng. J..

[10] Wu S., Bao J., Gao Y., Hu W., Lu Z. (2024). Review on flexible radiation-protective clothing materials. J. Mater. Sci..

[11] Du X., Jiang S., Zhao Q., Zhu M., Wang H., Wang C., He A., Ye X., Zhao B. (2023). Studies on aging behavior and degradation mechanism of raw EPDM rubber under γ-Ray irradiation. J. Compos. Biodegrad. Polym..

[12] Dou R., Zhang Y., Huang Z., Liu Q., Huang W., Meng X., Chen H. (2024). Study on the aging behavior and mechanism of nitrile rubber composites in combined radiation-thermal environments. J. Anal. Appl. Pyrolysis.

[13] Zeng C., Kang Q., Duan Z., Qin B., Feng X., Lu H., Lin Y. (2023). Development of polymer composites in radiation shielding applications: A review. J. Inorg. Organomet. Polym. Mater..

[14] Bagheri S., Khalafi H., Tohidifar M.R., Bagheri S. (2024). Thermoplastic and thermoset polymer matrix composites reinforced with bismuth oxide as radiation shielding materials. Compos. Part B Eng..

[15] Kalkornsuranee E., Intom S., Lehma N., Johns J., Kothan S., Sengloyluan K., Chaiphaksa W., Kaewkhao J. (2020). Mechanical and gamma radiation shielding properties of natural rubber composites: Effects of bismuth oxide (Bi_2_O_3_) and lead oxide (PbO). Mater. Res. Innov..

[16] Li Y., Liu F., Liu X., Li L., Kang Z., He Y. Study on performance of new flexible shielding materials and first demonstration application in CPR nuclear power plant. Proceedings of the International Conference on Nuclear Engineering.

[17] Erkoyuncu İ., Demirkol İ., Akman F., Dilsiz K., Kaçal M., Polat H. (2024). A detailed investigation of gamma and neutron shielding capabilities of concrete doped with bronze and boron carbide. Radiat. Phys. Chem..

[18] Nam H., Nam J., Yoo J., Kim S., Kang K. (2025). Radiation-induced degradation of butyl rubber: Impact on protection against chemical warfare agents. Radiat. Phys. Chem..

[19] Lee J., Yadav A., Antia M., Zaffino V., Flitsiyan E., Chernyak L., Salzman J., Meyler B., Ahn S., Ren F. (2017). Low dose ^60^Co gamma-irradiation effects on electronic carrier transport and DC characteristics of AlGaN/GaN high-electron-mobility transistors. Radiat. Eff. Defects Solids.

[20] Salama E., El-Khateeb S.A. (2022). The effect of gamma irradiation on the electrical and mechanical properties of bismuth carbonate-silicone rubber. Radiat. Eff. Defects Solids.

[21] Wang X., Yang K., Zhang P. (2022). Evaluation of the aging coefficient and the aging lifetime of carbon black-filled styrene-isoprene-butadiene rubber after thermal-oxidative aging. Compos. Sci. Technol..

[22] Wu Z., Zhen C., Huang C., Wang X., Wang X. (2025). A Study on the effects of multiple factors on the irradiation aging of EPDM composites: Experimental and machine learning approaches. J. Appl. Polym. Sci..

[23] Zaghdoudi M., Kömmling A., Jaunich M., Wolff D. (2019). Scission, cross-linking, and physical relaxation during thermal degradation of elastomers. Polymers.

[24] Lou W., Xie C., Guan X. (2022). Understanding radiation-thermal aging of polydimethylsiloxane rubber through molecular dynamics simulation. Npj Mater. Degrad..

[25] Luo R., Kang D., Huang C., Yan T., Li P., Ren H., Zhang Z. (2023). Mechanical properties, radiation resistance performances, and mechanism insights of nitrile butadiene rubber irradiated with high-dose gamma rays. Polymers.

[26] Hanada S., Miyamoto M., Hirai N., Yang L., Ohki Y. (2017). Experimental investigation of the degradation mechanism of silicone rubber exposed to heat and gamma rays. High Volt..

[27] Marinović-Cincović M., Marković G., Samaržija-Jovanović S., Budinski-Simendić J., Jovanović V. (2013). The influence of γ radiation on the properties of elastomers based on ethylene propylene diene terpolymer and chlorosulfonated polyethylene rubber. J. Thermoplast. Compos. Mater..

[28] Xiao Y., Zhang F., Wei R., Qin D., Tang Z., Bao Y., Cai Z. (2024). Influence of γ-irradiation dose on the mechanical and tribological properties of fluoroelastomer. Polym. Eng. Sci..

[29] (2021). Plastics—Determination of Water Vapor Transmission Rate.

[30] (2018). Plastics—Determination of Tensile Properties.

[31] (2009). Rubber, Vulcanized or Thermoplastic-Determination of Tensile Stress-Strain Properties.

[32] (2013). Determination of Tensile Properties of Plastics—Part 1: General Principles.

[33] Masoud T., Eesaee M., Hassanipour M., Elkoun S., David E., Nguyen-Tri P. (2025). Thermal aging behavior and lifetime prediction of industrial elastomeric compounds based on styrene–butadiene rubber. Polym. Eng. Sci..

[34] Gillen K.T., Bernstein R., Clough R.L., Celina M. (2006). Lifetime predictions for semi-crystalline cable insulation materials: I. Mechanical properties and oxygen consumption measurements on EPR materials. Polym. Degrad. Stabil..

[35] Celina M., Gillen K.T., Wise J., Clough R. (1996). Anomalous aging phenomena in a crosslinked polyolefin cable insulation. Radiat. Phys. Chem..

[36] Sheehan C.J., Bisio A.L. (1966). Polymer/Solvent interaction parameters. Rubber Chem. Technol..

